# 

**Published:** 1907-02

**Authors:** 


					﻿Sixty-second Year.
Vol. LXII. FEBRUARY, 1907.	No. 7
BUFFALO
MEDICAL JOURNAL
Established 1845 bv AUSTIN FLINT. M. D.
EDITOR:
WILLIAmjVARREN POTTER,
ASSISTANT EDITORS:
״j^JLjLIAM C. KRAUSS, M. E	NELSON W. WbLSZIS, M. D.
__________________ASSOCIATE EDITORS;
JAMES WRIGHT PUTNAM, M. D. ERNEST WENDE, M. D.
JOHN PARMENTER, M. D.	JOHN A. MILLER, Ph. D.
HARVEY R. GAYLORD, M. D.	MAUD J. FRYE, M. D.
JOHN A. RAFTER, M. D.
				

